# The moderating role of neighborhood social cohesion in the mediation effects of the loneliness between acculturation stress and post-traumatic growth among female North Korean defectors

**DOI:** 10.1038/s41598-023-43741-3

**Published:** 2023-10-08

**Authors:** Hokon Kim, Ocksim Kim, Kyoung-A Kim, Sang Hui Chu, Misook L. Chung

**Affiliations:** 1 College of Nursing and Brain Korea 21 FOUR project, Yonsei University, Seoul, Republic of Korea; 2 Department of Nursing, Suwon Women’s University, Suwon, Republic of Korea; 3 College of Nursing, Yonsei University, Seoul, Republic of Korea; 4 Mo-Im Kim Nursing Research Institute, College of Nursing, Yonsei University, Seoul, Republic of Korea; 5 College of Nursing, University of Kentucky, Lexington, KY USA

**Keywords:** Psychology, Health care, Risk factors

## Abstract

Loneliness negatively predicts post-traumatic growth (PTG) among North Korean defectors (NKD), one of the representative groups of refugees. Additionally, evidence also suggests that females, who account for 70% of NKDs entering South Korea, are vulnerable not only to past trauma but also to the current acculturation stress and loneliness affected by neighborhood social cohesion. This study explores whether the mediating effect of loneliness on the relationship between acculturation stress and PTG was moderated by the neighborhood social cohesion among female NKDs. In this study, the data of 166 female NKDs who completed an online survey regarding acculturation stress, PTG, loneliness, and neighborhood social cohesion were used. Moderated mediation analysis was conducted using SPSS PROCESS macro program. Loneliness was associated with PTG (B = − 1.896,* p* < 0.001), and mediated the association between acculturation stress and PTG (indirect effect = − 0.278, 95% LLCI − 0.403, 95% ULCI − 0.166). Neighborhood social cohesion moderated the mediation effect of loneliness on the association between acculturation stress and PTG (B = − 0.016, 95% LLCI 0.001, 95% ULCI 0.035). The indirect effect of acculturation stress on PTG through loneliness was notably high for those with low neighborhood social cohesion. Therefore, increasing neighborhood social cohesion would reduce loneliness caused by acculturation stress and support the positive growth among female NKDs. This represents the most effective approach to aiding female NKDs in achieving growth, even after suffering trauma.

## Introduction

By the year 2021, approximately 33,000 North Korean defectors (NKDs), a group that characterizes refugees and immigrants, had entered South Korea, driven by reasons such as seeking freedom from the oppressive regime's surveillance and control, facing food shortages, and striving to provide their families with better living conditions, of which more than 70% comprise females^1^. The prevalence of female NKDs has notably increased, surpassing that of male^2^ as they have comparatively easier movement between regions and access to border areas in neighboring countries such as China^3^. Generally, NKDs have been exposed to extreme trauma such as witnessing other’s deaths, experiencing life-threatening poverty, inhuman treatment, and the fear of repatriation to North Korea in the process of escaping from North Korea. Additionally, female NKDs are exposed to even sexual violence and prostitution in neighboring countries due to the unstable illegal immigrant state before entering South Korea^3–5^. Throughout this process, female NKDs face higher vulnerability to physical and mental violence as well as discrimination, compared to male NKDs^4^. A higher prevalence of post-traumatic stress disorder (PTSD) was also shown among female NKDs, than among male NKDs^6^.

Early PTSD studies have also confirmed that females were more likely to develop PTSD as twice than males^7^. Similarly, a study involving earthquake victims in China reported that females were more affected by traumatic experiences, showing a gender difference in the incidence of PTSD as well as in the level of post-traumatic growth (PTG)^8^. Another study of US troops deployed in Afghanistan and Iran reported that traumatic experiences and PTSD symptoms were more strongly correlated in females^9^. It is of upmost importance to prioritize research on female NKDs in South Korea, recognizing their distinct experience of trauma and PTSD.

In previous studies on refugees, the primary focus was on trauma experience and their negative impact on mental health, such as PTSD and complex PTSD^10,11^. However, there has been a recent shift towards acknowledging positive change and growth resulting from overcoming trauma^12^. As a new perspective on traumatic experiences, PTG is defined as a positive psychological change experienced as a result of the struggle with highly challenging life circumstances^13^. Not all NKDs came to South Korea. After escaping North Korea, the rate of successful completion of the journey to South Korea was under 20% of all NKDs^3^. Female NKDs who arrived in South Korea were people who had overcome past hardships and moved forward. Thus, PTG is a suitable variable to show their characteristics and to be able to confirm the positive direction of their overcoming and growth. In our previous study, we found that the PTG was significantly positively correlated with the current quality of life among 212 NKDs; in contrast, loneliness were strongly negatively correlated with PTG^14^.

There are controversial opinions on the effect of traumas on PTG depending on the severity and types of traumas experienced. According to a recent study of NKDs in South Korea, major traumatic events during the defection process continue to influence their current lives. Yet, the challenges of unexpected adaptation after arriving in South Korea and trauma resulting from discrimination-related issues had a more significant impact^15^. These on-going difficulties in social relationship were associated with complex PTSD and might pose a substantial obstacle to their continuous adaptation to an entirely different society.

Even though South and North Korea shared the same history and language, they have developed into completely different societies, economies, and cultures since Korea was divided into the South and North after the Korean War, more than 70 years ago^16^. North Korean society, specifically, is the world’s most closed and state-controlled communist country. Therefore, when female NKDs first faced the ideology of capitalism, everyday terms mixed with those of various western cultures, different intonations, and the application of high technology to everyday life in South Korea, they experienced cultural shocks^17^. Considering these findings, understanding the association between post-migration difficulties (e.g. acculturation stress, unemployment, weak social network, and discrimination) and PTG becomes crucial in illuminating the coping mechanism and growth of female NKDs during their settlement journey in South Korea. However, research findings on the relationship between post-migration stressors and PTG can also vary depending on refugee populations. During the post-migration settlement process, weak social network showed a negative correlation with PTG in a refugee background in Norway^18^. On the contrary, post-migration stressors were exhibited positive association with PTG in Syrian refugees^19^. Therefore, it is worth considering a study focused on the NKDs, who predominantly consist of women, to explore this dynamic further.

Acculturation stress, defined as the stress caused by sudden and excessive changes experienced by an individual or group while adapting to a new culture, is related to cultural adaptation in complex ways; such adaptation is a multidimensional change process that involves behavior, values, and attitudes^20^, and also, is closely associated with mental health issue such as depression, anxiety, and loneliness^21,22^. In particular, female NKDs experience greater mental problems related acculturation stress compared to their male counterparts^23^. This is due to their increased responsibility and burden of providing for the family’s economic income, as they are more likely to work in low-income job fields. Additionally, they also bear the responsibility of taking care of children who migrated with them^24^.

This study intended to explore how acculturation stress affects PTG and the role of loneliness and neighborhood social cohesion in female NKDs. Loneliness is a mental cognitive state that is differentiated from isolation; it comprises relational deficiencies, subjective evaluations, painful emotions, and contextual elements among the subjective emotions felt by individuals^25^. Research on immigrants in new societies with different cultures has consistently demonstrate that the acculturation stress is a significant predictor of increased feeling of loneliness^26,27^. For instance, a study focusing on migrant Chinese older adults revealed that acculturation had a negative direct effect on loneliness^28^. Recent studies on refugees have highlighted that loneliness exert a significant influence on their negative psychological well-being^29–32^. While substantial evidence exerts on mediating role of loneliness between stress and negative psychological well-being in various population^33,34^, there is limited knowledge regarding loneliness in female NKDs in South Korea^14^. Thus, it is necessary to examine how loneliness mediates negative impacts of acculturations stress on the PTG specifically among female NKDs in South Korea.

Neighborhood social cohesion shares some similarities with an individual’s social network, and denotes the network of relationships, shared values, and norms of residents within a neighborhood^35^. It is an indicator of the extent to which individuals interact with their neighbors and how neighbors perceive each other^36^. Immigrants’ neighbors play an important role in interventions against loneliness and cultural adaptation; enhancing the support and cohesion of such neighbors decreases the loneliness of immigrants and increases their adaptation to new communities^37,38^. Research on acculturation stress and psychosocial adjustment of youth from multicultural families in South Korea confirmed the importance of the neighbor role^39^. Emotional, informational, and material support received through neighbors and the community by migrant workers in South Korea was reported to be effective in reducing acculturation stress^40^. Although acculturation stress, or post-migration stress, is associated with PTG in populations with a refugee background^18,19,41^, there is still limited knowledge of how loneliness and neighborhood social cohesion affect the association between acculturation stress and PTG^36,42^. Therefore, it is worthwhile to assess the level of neighborhood social cohesion and its association with acculturation stress and loneliness among female NKDs. Based on the reviewed literature, we tested the relationship among acculturation stress, PTG, loneliness, and neighborhood social cohesion.

Hence, this study (1) examined whether acculturation stress would be related to PTG directly and indirectly mediated by loneliness, and (2) determined whether the mediation effect of loneliness on the association between acculturation stress and PTG was moderated by neighborhood social cohesion.

## Methods

### Study design and sample

The present study is based on data collected in our previous online survey in 2020, which was approved by the Institutional Review Boards of Yonsei University Health System (Y-2020-0019) in South Korea^14^ and was conducted in accordance with relevant regulations and guidelines. Informed consent was obtained from all participants before they answered questions in the online survey. The details on participants, recruitment, and data collection are described in the previous study^14^. The current analysis used the data of 166 female NKDs who provided complete data, including responses detailing acculturation stress, PTG, loneliness, and neighborhood social cohesion.

### Measures

#### Post-traumatic growth

PTG was measured using the Korean version of Post Traumatic Growth Inventory (PTGI), which assesses the degree of growth after traumatic experiences. The initial PTGI consisted of 21 items that were grouped into five factors (personal strength, new possibilities, improved relationships, spiritual growth, and appreciation for life)^13^. The Korean version of PTGI was ultimately revised to 16 items grouped into four factors (changes in self-perception, increase in interpersonal depth, finding new possibilities, and the increase in spiritual interest) after conducting a factor analysis^43^. The 16 items of the scale are rated on a 6-point Likert scale ranging from 0 to 5. The total score is calculated by summing all item scores. The possible score range is 0–80 points, with higher scores indicating more positive post-traumatic changes. The internal consistency of the scale was high in this study (Cronbach’s α = 0.932).

#### Acculturation stress

Acculturation stress was assessed using the Acculturation Stress Scale that was adapted for NKDs^44^. The 33 items of the Acculturation Stress Scale-NKD assess stress from perceived discrimination (six items), homesickness (four items), perceived hate (five items), fear (four items), culture shock (three items), guilt (two items), and nonspecific concerns (nine items) on a 4-point scale from 1 (strongly disagree) to 4 (strongly agree). The total score is calculated by summing all item scores; higher scores indicate greater acculturation stress. The internal consistency of the scale was high in this study (Cronbach’s α = 0.933).

#### Loneliness

Loneliness was measured using the 6-item Revised UCLA Loneliness Scale (RULS-6), revised by Neto in 2014 and validated by Wongparkarn in 2020^25,45,46^. The RULS-6 assesses self-perceived loneliness through feelings experienced in six situations, using a 4 point scale ranging from 1 (strongly disagree) to 4 (strongly agree). Higher total scores indicate higher degrees of loneliness. The internal consistency of the scale was high in this study (Cronbach’s α = 0.843).

#### Neighborhood social cohesion

Neighborhood social cohesion was assessed using the Neighborhood-Level Cohesion and Disorder Scale^47^. The original scale comprised two dimensions with eight items. We used the four items of the social cohesion dimension from the original measurement to confirm the level of neighborhood-level cohesion surrounding the participants (e.g., “I really feel part of this area; most people in this area can be trusted; most people in this area are friendly; if you were in trouble, there are lots of people in this area who would help you”). Higher total scores indicate higher levels of neighborhood social cohesion. Internal consistency of the scale was high in this study (Cronbach’s α = 0.822).

We also collected data of demographic characteristics to describe female NKD, namely age, marital status, education in North Korea, religion, employment status, monthly income in South Korea, living with family, the year of escape from North Korea, years spent in South Korea, and duration of stay in any third country.

### Data analysis

Descriptive statistics (such as means, standard deviations, frequencies, and percentiles) were used to summarize participants’ characteristics. Bivariate relationships between continuous variables were evaluated using Pearson’s correlation coefficient. We performed a moderated mediation analysis using the SPSS PROCESS macro, version 4.0 (Model 7)^48^. Loneliness was considered as a mediator between acculturation stress and PTG, while neighborhood social cohesion was considered as a moderator between acculturation stress and loneliness. We examined direct, indirect, and interaction effects using bias-corrected bootstrapping in a regression^48^ after controlling for covariates identified as factors related to PTG in a previous research^14^, such as religion, living with family, years spent in South Korea, education in North Korea, monthly income in South Korea, and employment status. All data analyses were performed using SPSS 26.0 (IBM SPSS Inc, Armonk, New York, USA). The PROCESS macro computed and assessed interactions to determine whether the direct and indirect effects were conditional on different levels of the moderator (i.e., neighborhood social cohesion). The PROCESS also provided a specific index of moderated mediation. A statistically significant index of moderated mediation confirmed the presence of a true moderated mediation effect among acculturation stress and PTG.

## Results

### Sample characteristics

The mean age of female NKD was 36.8 years (SD = 10.3), and 45.8% were married. Most lived with family (78.3%) and had received an education higher than junior college in North Korea (28.3%). They were predominately religious (55.4%) and employed in South Korea (58.4%), with a monthly income of less than 2000 US Dollars (59.0%). The average duration of their emigration from North Korea was 14.1 years (SD = 6.3) as of 2020. Furthermore, 54.2% and 28.5% of the participants had been staying in South Korea for $\ge$ 10 and $<$ 5 years, respectively. In addition, 39.8% had stayed in third countries for $\le$ 1 year before arriving in South Korea (Table 1).

**Table 1:** Characteristics and PTG comparison according to demographic characteristics of female North Korean defectors (*N* = 166).

Characteristics		*N* (%)	M (SD)	PTG		
				M (SD)	F/t	*p*
Age (years)			36.8 (10.3)			
Marital status	Married	76 (45.8)		54.13 (16.61)	0.822	0.441
	Single	44 (26.5)		50.45 (14.40)		
	Other	46 (27.7)		51.98 (14.83)		
Living with family	Yes	130 (78.3)		53.17 (15.45)	− 0.959	0.339
	No	36 (21.7)		50.36 (15.92)		
Education in North Korea	Elementary	26 (15.7)		46.16 (14.16)	2.792	0.064
	High school	93 (56.0)		53.30 (16.01)		
	College or higher	47 (28.3)		54.64 (14.72)		
Religion	Yes	92 (55.4)		56.33 (15.94)	3.604	< 0.000
	No	74 (44.6)		47.88 (13.77)		
Employment status	Employment	152 (91.6)		53.55 (15.40)	− 2.744	0.007
	Unemployed	14 (8.4)		41.86 (13.42)		
Years spent in South Korea	Under 5 years	34 (28.5)		49.59 (16.59)	0.779	0.460
	5–10 years	42 (25.3)		53.29 (15.01)		
	Over 10 years	90 (54.2)		53.34 (15.43)		
Monthly income in South Korea	None	48 (28.9)		50.83 (15.89)	1.496	0.227
	Under 2000 $	98 (59.0)		52.32 (15.81)		
	Over 2000$	20 (12.0)		57.90 (12.66)		
Acculturation stress			72.77 (17.69)			
Loneliness			12.45 (4.22)			
PTG			52.56 (15.55)			
Neighborhood social cohesion			11.80 (3.43)			

*§PTG* = Post-traumatic growth.

### Acculturation stress, loneliness, neighborhood social cohesion, and PTG

The mean acculturation stress score was 72.77 (SD = 17.69) out of a total of 132 points. The mean of each subcategory was: 8.78 (SD = 1.51) for culture shock, 10.02 (SD = 3.18) for homesickness, 14.2 (SD = 4.23) for perceived discrimination, 4.26 (SD = 1.71) for guilt, 8.47 (SD = 2.01) for fear, 10.0 (SD = 3.04) for perceived hate, and 22.73 (SD = 4.59) for nonspecific concerns. The mean PTG score was 52.36 (SD = 15.55) out of 80; a moderate level of PTG was therefore confirmed. The mean loneliness and neighborhood social cohesion score were 12.45 (SD = 4.22) and 11.80 (SD = 3.43), respectively (Table 1). The bivariate correlation analysis showed that acculturation stress had a significant positive relationship with loneliness (r = 0.619, p < 0.001) and negative relationships with PTG (r = − 0.227, p = 0.003) and neighborhood social cohesion (r = − 0.422, p < 0.001). Loneliness also had negative relationships with PTG (r = − 0.501, p < 0.001) and neighborhood social cohesion (r = − 0.364, p < 0.001). Finally, there was a positive relationship between PTG and neighborhood social cohesion (r = 0.353, p < 0.001) (Table 2).

**Table 2:** Pearson’s correlations of age, acculturation stress, loneliness, PTG, neighborhood social cohesion (*N* = 166).

	1	2	3	4	5
1. Age (years)	1				
2. Acculturation stress	0.108	1			
3. Loneliness	0.094	0.619**	1		
4. Neighborhood social cohesion	0.074	− 0.422**	− 0.364**	1	
5. PTG	0.099	− 0.227**	− 0.501**	0.353**	1

§*p < 0.005; **p < 0.001; *PTG* = Post-traumatic growth.

**Figure 1:**
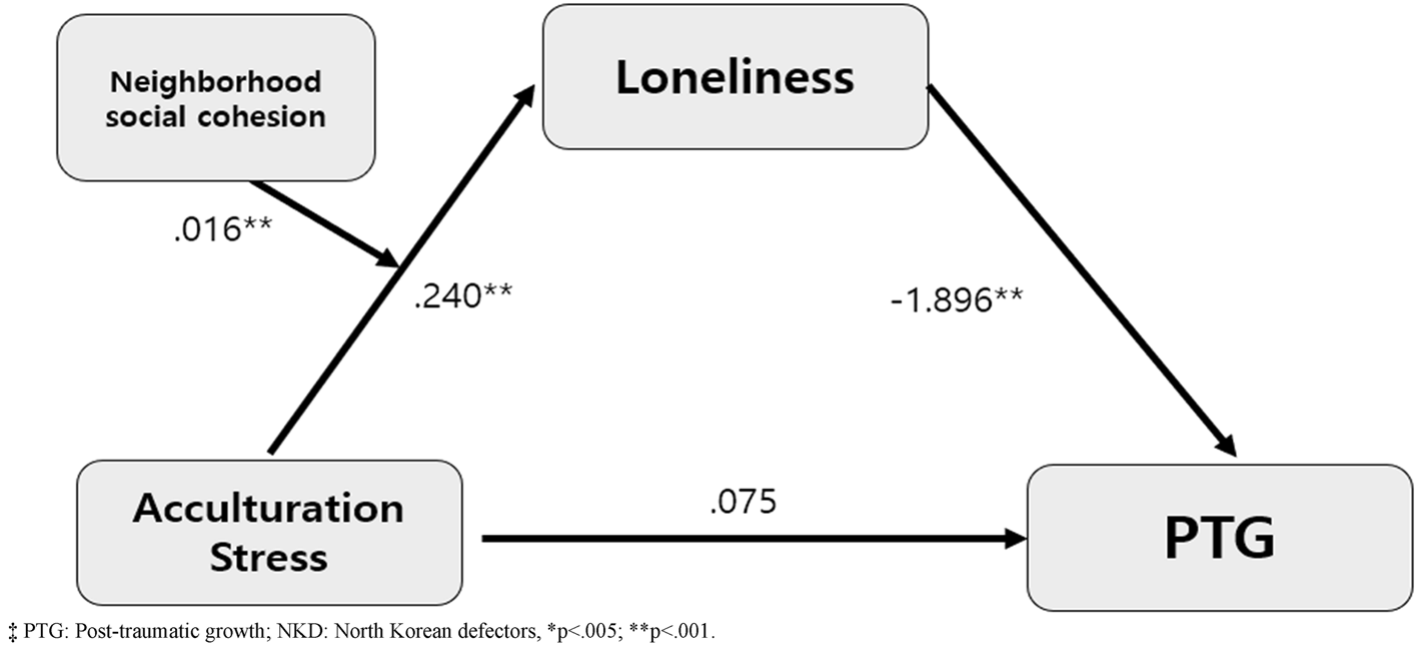
The moderation effect of neighborhood social cohesion in the mediation effect of loneliness between acculturation stress and post-traumatic growth in female North Korean defectors.

The mediation analysis confirmed the statistical significance of the total effect of acculturation stress on PTG (total effect = − 0.203, p = 0.003, 95% LLCI − 0.333, ULCI − 0.072). There waw no significant direct effect of acculturation stress on the PTG, but acculturation stress indirectly associated with PTG through loneliness (indirect effect = − 0.278, 95% LLCI − 0.403, ULCI − 0.166) (Table 3) (Fig. 1). It means that female NKDs who reported higher levels of acculturation stress also experienced greater feeling of loneliness, which, in turn, were associated with lower levels of PTG after controlling covariates, such as religion, living with family, years spent in South Korea, education in North Korea, monthly income in South Korea, employment status^14^.

**Table 3:** Moderated mediation effect of neighborhood social cohesion and loneliness on the association between acculturation stress and PTG with (Model 7 and Model 4).

Path and effect	B	SE	*t*	95% LLCI	95% ULCI	Adj R^2^	*p*
Acculturation stress to loneliness	0.240	0.048	4.987	0.145	0.335	0.487	< 0.001
Loneliness to PTG	− 1.896	0.325	− 5.828	− 2.538	− 1.253	0.351	< 0.001
Acculturation stress to PTG	0.075	0.077	0.984	− 0.076	0.227		0.327
Direct effect	0.075	0.077	0.984	− 0.076	0.227		0.327
Indirect effect	− 0.278	0.061		− 0.403	− 0.166		
Total effect	− 0.203	0.066	− 3.070	− 0.333	− 0.072		0.003
Moderated mediation effect	0.016	0.009		0.001	0.035		
Low NSC	− 0.325	0.071		− 0.472	− 0.192		
Moderate NSC	− 0.260	0.053		− 0.371	− 0.161		
High NSC	− 0.211	0.051		− 0.319	− 0.118		

§Covariates were religion, living with family, years spent in South Korea, education in North Korea, monthly income in South Korea, employment status. *PTG* Post-traumatic growth, *SE* Standard error, *LLCI* Lower bound of confidence interval, *ULCI* Upper bound of confidence interval.

**Figure 2:**
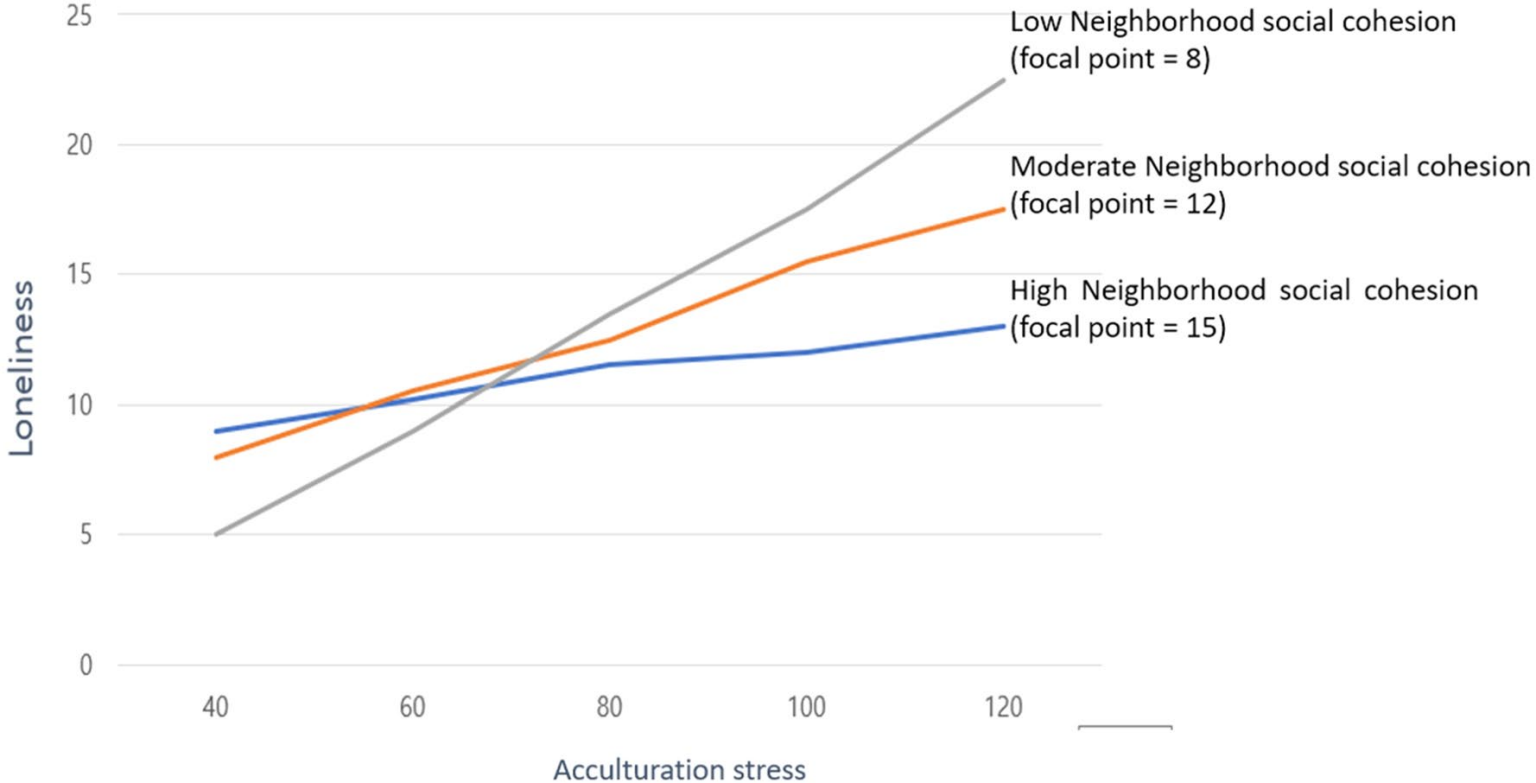
The moderated effect of neighborhood social cohesion on the association between acculturation stress and loneliness.

We found significant moderation effect of neighborhood social cohesion in the association between acculturation stress and loneliness (Fig. 2). The strength of the positive association between acculturation stress and loneliness was strongest among female NKDs who reported low neighborhood social cohesion. In contrast, for those who reported high level of neighborhood social cohesion, the strength of the association was comparatively weaker.

The mediation effect of loneliness in the relationship was founded to be significantly moderated by the levels of neighborhood social cohesion (moderated mediation effect = 0.016, 95% LLCI 0.001, ULCI 0.035). Among female NKDs, the indirect effect of acculturation stress on PTG through loneliness was notably high for those who reported low levels of neighborhood social cohesion (indirect effect = − 0.325, 95% LLCI − 0.472, ULCI − 0.192). In contrast, the indirect effect of acculturation stress on PTG through loneliness was considerably low for those who reported high levels of neighborhood social cohesion (indirect effect = − 0.211, 95% LLCI − 0.319, ULCI − 0.118).

## Discussion

In this study, we showed that significant mediating role of loneliness between acculturation stress and PTG. Moreover, we found that neighborhood social cohesion played a crucial moderating role in the mediation effect of loneliness in the association between acculturation stress and PTG in female NKDs. This study provides insight into the potential of community-based approaches to support female NKDs who, as refugees and immigrants, experience acculturation stress and loneliness in South Korea.

As anticipated, acculturation stress was strongly associated with loneliness in female NKDs. Similar to the current results, a study on Portuguese immigrants in Luxembourg also reported acculturation stress, specifically cultural identity conflict as a strong predictor of perceived loneliness^49^. Female NKDs who reported higher levels of acculturation stress also experienced greater feeling of loneliness, which, in turn, were associated with lower levels of PTG in this study. However, there exists contradictory research findings on the relationship between loneliness and PTG. For instance, a study involving war veterans revealed that higher levels of PTG could paradoxically correlate with increased feelings of loneliness, explained by increased realization of a civilian-military gap. Moreover, the study found that higher level of PTG consistently predicted higher loneliness over time whereas loneliness didn’t predict PTG in war veterans^50^. Yet, the experiences of war veterans and NKDs can lead to different forms of loneliness. War veterans may feel a sense of loneliness due to their changed subjective views of reality and the emotional gap between them and civilians caused by the traumatic experiences of war when they return to their original community. On the other hand, North Korean defectors experience loneliness in a new society where they lack social networks, as they have traumatic experiences and are separated from their familiar social connections. Both groups face distinct paths to experiencing loneliness based on their unique backgrounds and challenges. Understanding and respecting these differences is crucial to resolve the relationship between loneliness and PTG.

Contrary to our initial expectations, acculturation stress does not exert a direct impact on PTG among female NKDs. Female NKDs may undergo acculturation stress and PTG in different ways from male NKDs or other refugee populations due to the unique traumatic events they have endured and the challenges they face. Prior research indicates that female NKDs frequently encounter gender-specific forms of trauma, including sexual violence, which may result in additional challenges as they adapt to a new culture and society^3,5^. For the cultural adaptation of refugees and immigrants, language issues have been reported as the most significant source of acculturation stress. In a study focused on Koran American immigrant populations, findings indicated that women experienced greater levels of acculturation stress compared to men, with less proficient in English than men^51^. While the acculturation of refugees and immigrants often involves language-related issues that contribute significantly to acculturation stress, this scenario might differ for female NKDs, who share the Korean language. Nevertheless, the additional burden of traditional caregiving responsibilities typically assigned to women, coupled with economical responsibilities due to the easier job opportunities than men in new societies^3,52^, seems to have contributed to an increased sense of loneliness for female NKDs who lack social resources and networks^30^. In particular, South Korean society is known for experiencing higher levels of loneliness when individuals do not belong to similar groups or lack a sense of community cohesion^53,54^. Particularly, during the process of raising children, female NKDs often encounter emotional challenges due to inadequate understanding of the highly competitive and academically driven educational environment, weak connections with other fellow parents, and a lack of support resources^55^. Consequently, it is crucial to carefully account for the distinctive experiences of female NKDs when interpreting the present data.

To the best of our knowledge, this is the first study to reveal a notable moderating effect of neighborhood social cohesion on the relationship between acculturation stress and loneliness. Low neighborhood social cohesion amplified the positive link between acculturation stress and loneliness for female NKDs, while high cohesion weakened this association. Our study suggests that female NKDs could benefit significantly from social support within local and neighboring communities. Increasing the level of neighborhood social cohesion emerged as a key strategy to improve several dimensions of refugee life through improving daily activities, psychological states, and other health-related indicators^35,56^. To advance neighborhood social cohesion, the cooperation of community resources, informal networks, and multidisciplinary expert groups including psychiatry, nursing, and psychology is required^57^. In particular, based on understanding the characteristics of the original community culture, establishing a direct social network through multidisciplinary cooperation was identified as an important step in building community for new refugees in Canada^58^. Similarly, female NKDs acquired psychological comfort through exchange with their local or neighboring communities and the associated social support^42^. A potential source of social support may be informal networks of family, friends, and neighbors. These individuals may offer practical assistance such as help with transportation or housing, as well as emotional support and a sense of belonging. In a qualitative research, NKDs initially acquired an emotional belonging and stability through forming the relationship with South Koreans^59^. Local religious or faith-based communities also may provide a supportive environment for female NKDs. Studies of the adaptation patterns of female NKDs to South Korean society have also shown that they cope with adaptation through the formation of internal communities as one of their strategies^17^.

For stable adaptation and growth as a member of the South Korean society for female NKDs, an interventional approach is needed to enhance neighborhood social cohesion with external neighbors or communities that form part of their own internal network resources. Even after intensive South Korean social adaptation program led by the Ministry of Unification in the early days of entry^17^, it is necessary to focus on forming a human network to bond with neighbors and communities during each settlement period^60^. The community-based organizations or support groups would provide a safe and supportive space for individuals to share their experiences and connect with others who have similar backgrounds. These groups may provide emotional support, practical assistance, and opportunities for socialization and community involvement. Therefore, it is crucial to develop programs and strategies that leverage the strengths of female NKDs and foster an environment where they can coexist and support one another in connection with community in religious organization, workplace, and school^60^. This can be achieved by harnessing the knowledge, assistance, and leadership skills of female NKDs who have undergone similar experiences^24^. Additionally, establishing an easily accessible mental health system capable of identifying and promptly addressing feelings of loneliness and other psychological issues among female NKDs is essential^61^. Facilitating emotional connections with new neighbors or communities can greatly contribute to the adaptation and personal growth of female NKDs in South Korea. Overall, there are many potential sources of social support for female NKDs, and the specific types of support that may be most beneficial may vary depending on individual needs and preferences. It is important for interventions and support programs to be tailored to the specific needs of this population and to take into account their unique experiences and challenges.

Although the findings of this research are valuable, this study has some limitations. First, the use of a cross-sectional approach in this study restricts the ability to establish causal relationships between acculturation stress, loneliness, and PTG over time. To address this, future research should adopt a longitudinal design to capture temporal associations accurately. Second, while the study focused on female NKDs in South Korea, the relatively small sample size of 166 participants might limit the generalizability of findings. A more diverse participant pool, including female NKDs from different geographical contexts (e.g. China, South East countries etc.), is necessary to broaden the study's applicability. Lastly, relying solely on quantitative methods might not fully capture the intricate aspects of PTG among female NKDs. A more comprehensive understanding can be achieved by incorporating qualitative methods, allowing for a deeper exploration of intrinsic factors like resilience and coping strategies.

In conclusion, our research emphasizes the importance of neighborhood social cohesion and its interplay with loneliness in shaping the relationship between acculturation stress and PTG among female NKDs. Cultivating a sense of neighborhood social cohesion holds a key role in alleviating the loneliness triggered by acculturation stress and in fostering the process of personal growth for female NKDs. This approach would stand as a potent avenue to empower female NKDs, enabling them to navigate growth and surmount the enduring impact of past traumas effectively.

## Data Availability

The datasets generated during and/or analyzed during the current study are available from the corresponding author on reasonable request**.**
